# Geothermal favourability in data-scarce regions: incorporating physical and socio-economic factors into a modified Play fairway approach, southwestern Yukon, Canada

**DOI:** 10.1186/s40517-025-00345-6

**Published:** 2025-05-16

**Authors:** F. M. Chapman, M. M. Miranda, S. Sternbergh, R. Soucy La Roche, J. Raymond

**Affiliations:** 1 INRS—Institut national de la recherche scientifique, 490 Rue de La Couronne, Québec, QC G1K 9A9 Canada; 2 Yukon Geological Survey, P.O. Box 2703 (K14), Whitehorse, Yukon Y1A 2C6 Canada

**Keywords:** Energy, Low-temperature, Remote, North, Burwash Landing

## Abstract

Geothermal energy could be used to reduce or replace diesel for heating in remote northern communities. Geothermal development has primarily focused on shallow, high-temperature resources, but interest in low-temperature and deep geothermal resource exploration has increased as energy costs and climate change policy have evolved. Here, we evaluated the low-temperature geothermal favourability in southwestern Yukon by adapting Play fairway analysis to data-scarce regions. Play fairway analysis is a spatial statistical tool that uses a layered data approach to model favourability and risk assessments for resource exploration. Previous Play fairway analyses concentrate on the physical aspects of geothermal favourability: heat, permeability, and fluid availability. This study presents an overview of potential direct and indirect physical parameters that could be used in a geothermal Play fairway analysis in data-scarce regions and introduces the importance of considering socio-economic data in the exploration phase. The socio-economic controls are grouped into quantitative and qualitative parameters that describe population trends and community interests. The framework presented is then applied to a Play fairway analysis for southwestern Yukon. Based on the physical and socio-economic analysis, there is interest in exploring geothermal potential along the Denali fault near Duke River to support the community of Burwash Landing.

**Supplementary Information:**

The online version contains supplementary material available at 10.1186/s40517-025-00345-6.

## Introduction

Global energy production is primarily reliant on non-renewable resources which have contributed to the increase of atmospheric greenhouse gas concentrations since the industrial revolution (starting in 1760s; IPCC, 2014). In Canada, remote communities typically rely on micro-grids powered primarily by diesel or natural gas (Miranda et al. 2020). Local geothermal development in these communities would decrease the dependence on diesel imports and offer a local, clean, renewable, and consistent energy source.

Previously, geothermal development was restricted to areas of high tectonic and magmatic activities with steep geothermal gradients (e.g. Flovenz and Saemundsson, 1993; Thomas 1986), but low-temperature geothermal resource exploration has become more common (Miranda et al. 2020; Raymond et al. 2015; Stefansson 2005; Davalos-Elizondo, 2023). Low-temperature settings are widely distributed and are the most abundant form of potential geothermal energy sources in Canada (Stefansson 2005). It is essential to identify the most favourable locations within these low-temperature settings to facilitate the development of geothermal energy production (Davalos-Elizondo, 2023).

Play fairway analysis is a spatial and geostatistical tool that has been used extensively in oil and gas exploration to identify areas of high resource potential and low exploration risk (Fraser and Gawthorpe 2003). In a given region, physical data that contribute to a resource reservoir is identified and converted to spatial data. This data is reclassified based on contribution to a resource reservoir, weighted based on parameter significance, and then superposed to produce a map of resource favourability. Recently, this method has been adapted and used as a tool to identify favourable geothermal areas in high-temperature regions with high-density data where electricity generation is the target (e.g. Ito et al. 2017; Lindsey et al. 2021; Siler and Faulds, 2013; Siler et al. 2017; Wang et al. 2021). However, physical data required to make informed decisions about local geothermal energy potential are limited in remote regions. This limitation not only makes physical favourability analysis challenging, but also leads to significant uncertainty. Here, we demonstrate the potential to use Play fairway analysis in remote and data-scarce areas where low-temperature geothermal resources could be developed for both direct use and electricity generation, while highlighting the limitations due to data scarcity.

Previous Play fairway analyses have concentrated solely on the physical parameters associated with geothermal favourability (e.g. Hinz et al. 2016; Lindsey et al 2021). However, it is critical to also consider socio-economic factors when evaluating the geothermal favourability of remote regions. Geothermal resources exploited for direct use (i.e. space heating) can be transported effectively under short distance, generally on the order of 1–5 km, and electricity should be generated locally to decrease power-grid construction costs (Chandrasekharam and Bundschuh 2008). Additionally, many remote communities have unique ways of life, energy demands, and social structures, all of which should be considered as of the exploration stage.

Wang et al. (2021) integrate a “realistic” parameter which includes Territory of National Park and population density. The national park delineation identifies areas where development would not be feasible, regardless of physical potential, and population density helps gauge where development would be most beneficial. These are promising first steps towards the integration of social parameters in Play fairway analyses. Herein, we expand on the integration of social parameters to include quantitative variables (population and mobility, and education and labour force), as well as qualitative variables (community openness to development and community goals and projections). Considering the social factors in the exploration stage ensures that potential development is in the best interest of the targeted user.

The objective of this research was to develop a holistic Play fairway analysis framework for exploration of low-temperature geothermal resources in data-scarce regions that considers both geological and socio-economic factors. The development of such approach would be useful for several isolated communities in northern Canada. We first identify and summarize physical parameters used in previous geothermal Play fairway analyses. We then demonstrate how to identify and apply these parameters in data sparse areas by applying the proposed framework to evaluate the physical geothermal favourability in southwestern Yukon, which hosts potential for low enthalpy geothermal systems. The relatively complex tectonic history of southwestern Yukon is suggestive of higher geothermal potential relative to other areas of this territory. The Play fairway analysis herein therefore identifies areas of increased favourability within southwestern Yukon. We finally highlight the importance of considering the socio-economic context around areas of physical geothermal favourability in the exploration stage by conducting a socio-economic analysis in favourable areas of southwestern Yukon. This approach is designed to identify the challenges associated with remote and data-scarce areas while providing insight into how to adapt traditional techniques to incorporate socio-economic analysis and be applicable in these regions.

## Background information

The Government of Yukon has committed to reducing greenhouse gas emission by 30% by 2030 (Government of Yukon, 2020), taking a lead role in climate action in Canada. Investment in geothermal energy in remote communities can aid the Government of Yukon to meet their emission goals by reducing diesel consumption in the territory.

### Southwestern Yukon

**Fig. 1:**
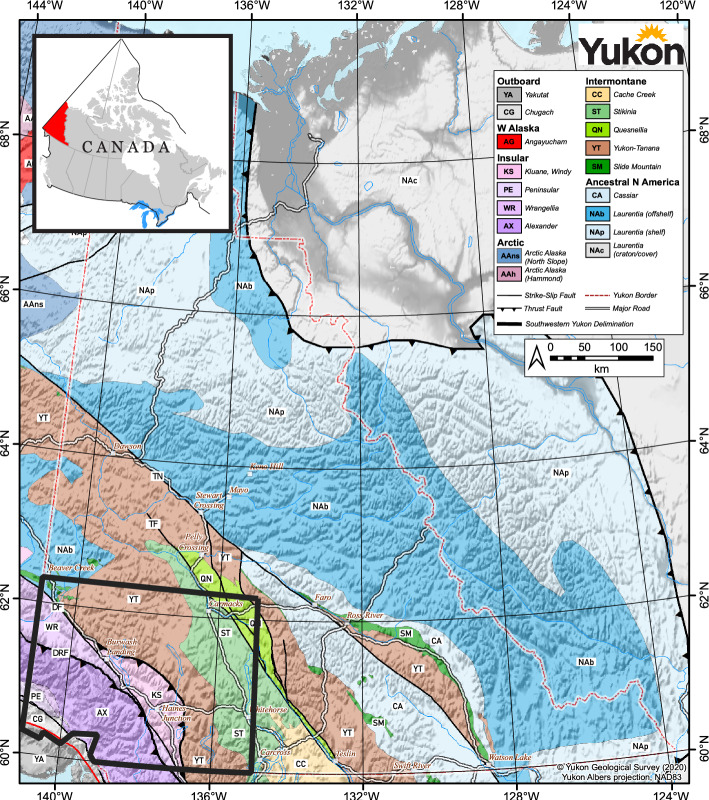
Surface terrane map of Yukon adapted from Yukon Geological Survey (2020) Terrane Map. The extent of Yukon is indicated by red-dashed line on the terrane map. Southwestern Yukon, the region of interest for the Play fairway analysis herein, is outlined in black. *DRF* Duke River Fault, *DF* Denali Fault, *TF* Teslin Fault, *TN* Tintina fault. Yukon is indicated by red fill on the inset map of Canada (adapted from Geomatics Canada, 2006)

Southwestern Yukon is located in the northern Canadian Cordillera and is bordered by the St. Elias Mountains to the southwest and the Teslin fault to the northeast (Fig. 1; Colpron et al. 2007). This area comprises, from northeast to southwest, Intermontane, Insular and Outboard allochthonous terranes, which are composed of units of variable origin and contrasting tectonic evolution (Colpron et al. 2007; Nelson and Colpron 2007). These include the Quesnellia, Stinkina, Yukon-Tanana, Kluane, Wrangellia, and Alexander terranes.

The Quesnellia and Stikinia terranes consist of late Paleozoic to Mesozoic juvenile island arc volcanic rocks and associated sedimentary rocks (Colpron et al. 2007; Nelson and Friedman 2004). The Yukon-Tanana terrane is exposed to the southwest and northwest of the Stikinia and Quesnellia, respectively (Fig. 1). The basement of the Yukon-Tanana terrane is the Late Devonian and older Snowcap assemblage, which shares properties with the Laurentian margin sedimentary units (Nelson et al. 2006). During the Carboniferous to Permian, a series of volcanic arcs developed onto the Snowcap assemblage resulting in the Finlayson, Klinkit, and Klondike assemblages (Colpron et al. 2007). The Quesnellia, Stikinia, and Yukon-Tanana terranes are all intruded by a Late Triassic-Early Jurassic plutonic suite (Nelson and Friedman 2004). The Kluane terrane is situated between the Insular and Intermontane terranes described herein. It is composed of Jurassic to Cretaceous basinal assemblages intruded by voluminous Paleocene to Eocene magmatic rocks (Colpron et al. 2016; Eisbacher 1976; Israel et al. 2011). The Wrangellia terrane is composed of Paleozoic and Mesozoic volcanic, sedimentary, and intrusive rocks (Israel et al. 2006). In southwestern Yukon, the Alexander terrane is composed of Cambrian to Devonian meta-sedimentary and meta-volcanic rocks intruded by Pennsylvanian to Early Permian plutons (Israel et al. 2006; Cobbett et al. 2017).

The distribution of Intermontane and Insular terranes in southwestern Yukon is controlled by faults and shear zones that formed during and after accretion. The Teslin, Denali, and Duke River faults are strike-slip faults separating these terranes. (Snyder et al. 2005). In the study area, the Teslin fault (TF; Fig. 1) separates the Quesnellia terrane and the Stikinia terrane (De Keijzer et al. 2000). The Denali fault (DF; Fig. 1) separates the Yukon-Tanana, Stikinia and Kluane terranes (northeast) from the Wrangellia and Alexander terranes (southwest; Choi et al. 2021; Nelson & Colpron 2007). The Wrangellia and Alexander terranes are separated by the Duke River fault (DRF; Fig. 1; Nelson and Colpron 2007).

The Teslin fault is confined to the upper crust and was active during the Late Cretaceous (De Keijzer et al. 2000; Snyder et al. 2005). The dextral Denali fault spans over 2 000 km from central Alaska, through southwestern Yukon, and into northern British Columbia (Choi et al. 2021; Nelson and Colpron 2007). The Denali fault has been active since the Cretaceous period. Today, the section in the study area, the eastern Denali fault, experiences less activity than the Alaskan portion of the Denali fault (Bender and Haeussler, 2017; Blais-Stevens et al. 2020). The present-day average slip rate is ~ 5 to 12 mm per year along the Alaskan portion of the Denali fault, compared to ~ 2 to 5 mm per year for the eastern Denali fault (Blais-Stevens et al. 2020; Haeussler et al. 2017; Leonard et al. 2007). Predominantly a strike-slip fault, a vertical displacement of 0.2 to 0.9 mm per year has also been reported in the eastern Denali fault (Marechal et al. 2018; McDermott et al. 2019).

The Duke River fault separates the Wrangellia terrane to the northeast from the Alexander terrane to the southwest (Choi et al. 2021; Nelson and Colpron 2007). Ductile displacement along the Duke River fault initiated in the Cretaceous (~ 145 to 66 Ma), but brittle displacement began around the Miocene (~ 23 Ma; Cobbett et al. 2017; Leonard et al. 2007). Present-day horizontal and vertical displacement along the Duke River fault has been estimated at ~ 3 mm and ~ 1.5 mm per year, respectively (Leonard et al. 2007).

### Geothermal systems

Geothermal energy can be harvested using open- or closed-loop geothermal systems. Geothermal power-plants and district heating systems primarily rely on open-loop well configurations (Zarrouk and Moon 2014; Moya et al. 2018) though new closed-loop technology for geothermal power plants has been developed (e.g. EavorLoop; Kelly and McDermott 2022). In an open-loop geothermal system, groundwater is used directly, and at least two wells are required, one for groundwater extraction and one for waste-water injection. Hot groundwater is extracted, and circulated to a geothermal plant where the heat is transferred to a heat carrier fluid by a heat exchanger. Through this process, the groundwater cools and is then reinjected into the same aquifer. Alternative open-loop systems may inject waste-water into a separate aquifer or surface water body. The efficiency of this system is highly dependent on flow-rate. To maintain a sustainable system, the rate of groundwater extraction must not exceed the rate at which groundwater is naturally replenished; therefore aquifer permeability and fluid availability have a significant influence on an areas favourability to operate an open-loop geothermal system.

In contrast, closed-loop systems do not directly interact with groundwater. Heat is transferred from the ground to a closed-loop system by conduction. The rate of heat transfer depends on the thermal conductivity of the sediments or bedrock into which the closed-loop system is installed (Dehkordi and Schincariol 2014). Therefore, the thermal properties have a greater influence on closed-loop geothermal favourability compared to open-loop geothermal favourability although the presence of groundwater may have an impact on the performance of the system.

Play fairway analyses for geothermal exploration are typically used to evaluate the potential for open-loop geothermal systems to generate electricity and are performed in high-data density areas. An extensive literature review of these studies led to the development of the suggested framework for geothermal Play fairway analyses in remote data-scarce areas. Through the literature review, we identified three essential categories used to classify physical parameters that influence the quality and type of geothermal resource: thermal properties, permeability, and fluid availability. The relative importance of each category (thermal properties, permeability, and fluid availability) can be adjusted within a Play fairway analysis depending on the intended development (open versus closed-loop systems).

## Methods

### Physical Play fairway analysis framework

The general framework developed for Play fairway analysis in data-scarce regions is:

- Step 1: Identify relevant physical data available in the region.
- Step 2: Convert spatial data into raster files.
- Step 3: Reclassify data and assign weights to each parameter.
- Step 4: Superpose parameters within each category.
- Step 5: Assign weights to each category.
- Step 6: Superpose categories.
- Step 7: Perform a sensitivity analysis.

Each of these steps are described below and applied to the data available for southwestern Yukon in the results section.

- Step 1: Identify data available in the region

The first step is to identify pertinent data that are available in the region. Tables 1, 2, and 3 provide a comprehensive overview of data that could be used in Play fairway analysis to identify favourable geothermal areas based on previous Play fairway analyses and knowledge of requirements for geothermal reservoirs. These tables include direct and indirect data and can be used as a reference to determine potential data to search for within the study area. Direct data provide accurate insight into local conditions but are typically point measurements. In data-scarce areas, point measurements can be infrequent and unreliably to assess regional favourability. Indirect measurements with higher spatial coverage must be used to complement direct data. Direct and indirect data may be available in scientific articles (e.g. Davies, 2013; Li et al. 2017; Colpron 2019), government databases (e.g. Natural Resources Canada, 2017, 2021; Yukon Geological Survey 2024), or industry reports. In data-scarce areas, global datasets with extensive spatial coverage (e.g. Curie-point depth) may be more readily available. Justification for the inclusion of, and a general description of parameters in Tables 1, 2, and 3 are provided in supplementary material 1. Individual parameter descriptions are pertinent when selecting parameters to include in regional Play fairway analysis based on type of geothermal system and data availability.

**Table 1:** Thermal parameters used in previous geothermal Play fairway analyses in high-data density areas and available for the current study (A)

Data	1	2	3	4	5	6	7	8	9	10	11	12	A
Geothermal parameters													
Heat flow		X				X					X		X
Temperature gradient	X	X	X		X	X	X	X			X		X
Groundwater temperature				x		X	X					X	
Curie point depth													X
Hot spring temperature	X								X	X			
Geothermometry and geochemistry	X	X	X	X	X	X	X			X	X	X	
Hydrothermal alterations								X	X			X	
Volcanic and tectonic features													
Rift zones and stage				X	X								
Volcanic vents (incl. calderas) or centres	X	X		X	X						X		
Volcanism (age/recency and type)						X			X				
Uplift rate										X			
Dike density					X						X		
Intrusive rocks/radiogenic heat production	X												X
Geophysical measurements													
Magnetotelluric (MT)		X		X	X			X				X	
Gravity gradients		X	X	X	X		X				X		
Resistivity anomaly													
Geochemical indicators													
Gamma ray dose rate											X		
Lithium concentration											X		
Boron concentration											X		
Subsurface characteristics													
Basement depth											X		
Crustal thickness			X								X		
Other parameters													
Surface topography (DEM)											X		

References: 1. Forson et al. (2015), 2. Nielson et al. (2015), 3. Hinz et al. (2016), 4. Ito et al. (2017), 5. Lautze et al. (2017), 6. Siler et al. (2017), 7. Faulds et al. (2018), 8. Poux & O’Brien (2020), 9. Lindsey et al. (2021), 10. Wang et al. (2021), 11. Holmes & Fournier (2022), 12. Olvera-García et al. (2023)

**Table 2:** Permeability parameters used in previous geothermal Play fairway analyses in high-data density areas and available for the current study (A)

Data	1	2	3	4	5	6	7	8	9	10	11	12	A
Tectonic setting and fault-related													
Structural setting			X			X	X		X			X	
Faults (age and length)/fault system		X	X	X	X		X	X	X	X	X	X	X
Lineaments (morphological or geophysical)												X	
Rift zone					X								
Shear stress	X												
Dilation strain rate	X		X							X			
Slip tendency	X		X			X	X						
Dilation tendency	X					X	X						
Fault displacement distribution and gradient	X												
Volcanic and hydrothermal features													
Calderas									X				
Hot springs									X			X	
Hydrothermal alterations									X			X	
Volcanic vent alignment		X										X	
Fluid entries								X					
Geophysical measurements and earthquake-related													
Earthquakes or seismicity			X	X		X	X	X	X	X	X	X	X
Magnetic anomaly/gradients		X									X		
Gravity anomaly/gradients		X	X	X	X		X				X		
Surface topography (DEM)											X		
Dike density											X		
Topographic gradient or elevation change										X	X		
Strain related													
Strain rate	X				X	X	X				X		
Tensile fracture density	X												

References: 1. Forson et al. (2015), 2. Nielson et al. (2015), 3. Hinz et al. (2016), 4. Ito et al. (2017), 5. Lautze et al. (2017), 6. Siler et al. (2017), 7. Faulds et al. (2018), 8. Poux & O’Brien (2020), 9. Lindsey et al. (2021), 10. Wang et al. (2021), 11. Holmes & Fournier (2022), 12. Olvera-García et al. (2023)

**Table 3:** Fluid parameters used in previous geothermal Play fairway analyses in high-data density areas and available for the current study (A)

Data	1	2	3	4	5	6	7	8	9	10	11	12	A
Hydrological parameters													
Water table depth/elevation											X		
Water table gradient											X		
Groundwater recharge				X	X								
Precipitation											X		
Surface waters													X
Geothermal and hydrological features													
Surface springs									X		X		
Hydrothermal alteration									X				
Geothermometry											X		
Geochemical indicators													
Lithium concentration											X		
Boron concentration											X		
Geomorphological features													
Salars and lagoons									X				
Drainage density											X		
Geophysical measurements													
Magnetotelluric (incl. resistivity)				X	X								

References: 1. Forson et al. (2015), 2. Nielson et al. (2015), 3. Hinz et al. (2016), 4. Ito et al. (2017), 5. Lautze et al. (2017), 6. Siler et al. (2017), 7. Faulds et al. (2018), 8. Poux & O’Brien (2020), 9. Lindsey et al. (2021), 10. Wang et al. (2021), 11. Holmes & Fournier (2022), 12. Olvera-García et al. (2023)

The Play fairway analysis performed herein is for the exploration of low-temperature geothermal resources, and the parameters used for the Play fairway analysis of southwestern Yukon depended on data availability. Thermal parameters are heat flow, temperature gradient, Curie-point depth, and radiogenic heat production (Table 1). Permeability parameters are faults (age and length) and earthquakes (Table 2). Fluid availability parameters are surface waters (Table 3).

- Step 2: Convert spatial data into raster files

All parameters must be converted into raster data irrespective of the initial data source or format. Herein, non-raster data were converted from the original file type (CSV point data, shapefile) to raster data using the ‘Point to Raster’ or ‘Polygon to Raster (Conversion)’ tools in ArcGIS.

- Step 3: Reclassify and assign weights to each parameter

Once converted to raster files, the data were reclassified using ‘Reclassify’ from the ‘Spatial Analyst’ tool in ArcGIS. Parameters cannot be directly compared or superposed as each parameter has a unique unit of measurement not associated with geothermal favourability. Therefore, parameters must be reclassified on a scale of 1 to 10 from least favourable (1) to most favourable (10). Play fairway analyses use either statistically driven or knowledge-driven approaches to reclassify parameters. For data-scarce regions, we propose a knowledge-driven approach, in which the reclassification is determined based on the range of regional values for each parameter and compared to parameter ranges in regions with previous geothermal exploration (Lindsey et al. 2021). Once parameters have been identified, Tables 1, 2, and 3, can help identify references with example classifications. Favourability is unique to each region. The conditions which may be the most favourable in one area may be least favourable elsewhere. Therefore, the reclassification must be adjusted based on the range of possible values in a given study area.

The significance of a dataset will fluctuate depending on the spatial coverage of available data. For example, direct measurements, such as temperature gradients, are considered more reliable thermal indicators than indirect indicators such as the Curie-point depth. However, the Curie-point depth layer may be weighted higher due to the extensive spatial coverage. Here, we use the data available in southwestern Yukon to outline the reclassification and weighting process. The same approach can be used with a different combination of available data layers.

- Step 4: Superpose parameters within each category

The weighted-sum approach is used to combine parameters within each category to develop favourability maps. The weighted-sum approach ensures each evidence layer is considered in the final model despite data limitations (Lindsey et al. 2021). To apply the weighted-sum approach, the reclassified and weighted raster data (step 3) are superposed within each category using the ‘rgdal’ and ‘raster’ packages in RStudio.

- Step 5–6: Assign weights and superpose categories

Each category influences the geothermal potential and therefore must also be combined using the weighted-sum approach. The relative weight of each category should consider spatial coverage, quality of data in each category, and geothermal system of interest. Favourability maps can be produced using the ‘rgdal’ and ‘raster’ packages in RStudio. A sensitivity analysis is recommended to note any variation in favourability based on category weights.

- Step 7: Perform a sensitivity analysis

A knowledge-driven approach is used to assign the weights to individual layers within each category (thermal, permeability, and fluid). This approach considers the significance and spatial distribution of each parameter. Sensitivity analysis is not applied to the weighting of parameters within the categories as it would neglect the relative distribution of the data. However, sensitivity analysis is performed across categories to assess the impact of varying the relative weights of the thermal, permeability, and fluid layers. This sensitivity analysis results in multiple favourability maps and provides insight into the influence of each data category on geothermal favourability at different sites.

### Socio-economic analysis

Socio-economic indicators describe the social and economic context of a community. Socio-economic data inform these requirements and can be divided into three categories: spatial, quantitative, and qualitative (Table 4). Spatial data, such as proximity to power grid infrastructure, is included directly on the maps generated in the physical Play fairway analysis. Quantitative data are used statistically to determine variables such as population size, mobility, and community energy demands, and qualitative data are used to describe social capacity and considerations (Table 4).

**Table 4:** Socio-economic parameters to identify suitability of a remote community for geothermal development

Category	Data	Data type
Spatial data	Community location	Coordinates
	Power-grid infrastructure	Vector file
Quantitative data	Population and mobility	
	Population	Census data
	Private dwellings	
	Median age	
	Mobility	
	Education and income	
	Highest education attainment (% population)	Census data
	Labour force	
	Occupation category	
	Energy needs and current production	
	Energy demand—electricity	Community energy report
	Energy demand—heating	
	Energy production—electricity	
	Energy production—heating	
Qualitative data	Community understanding and perspective	
	Openness to geothermal development	Informal discussions, questionnaires, and interviews
	Community goals and projections	

The spatial data narrow down the potential areas of interest such that only communities within an accessible distance of a favourable area for geothermal energy will be considered. A quantitative and qualitative socio-economic analysis can then be applied to the potential communities of interest to identify the best-fit community for future research and development.

The quantitative parameters adopted herein are based on the methods used by Chitsaz (2022) to evaluate the socio-economic context with respect to geothermal development in Fort Nelson, BC. The qualitative data focus on social acceptability of renewable energy and local geothermal development. Previous research has shown that communities are generally interested in using renewable energy to reduce greenhouse gas emissions, but are hesitant primarily due to a lack of knowledge with regard to different renewable technologies, including geothermal energy (Dowd et al. 2011; Payera 2018; Pellizzone et al. 2016; Shamsuzzoha et al. 2012; Soltani et al. 2021). In certain communities, members have also expressed concern about the environmental impacts of geothermal energy (Dowd et al. 2011; Payera 2018).

## Results

The general framework described for data-scarce regions was applied to southwestern Yukon.

### Data available in southwestern Yukon

**Fig. 2:**
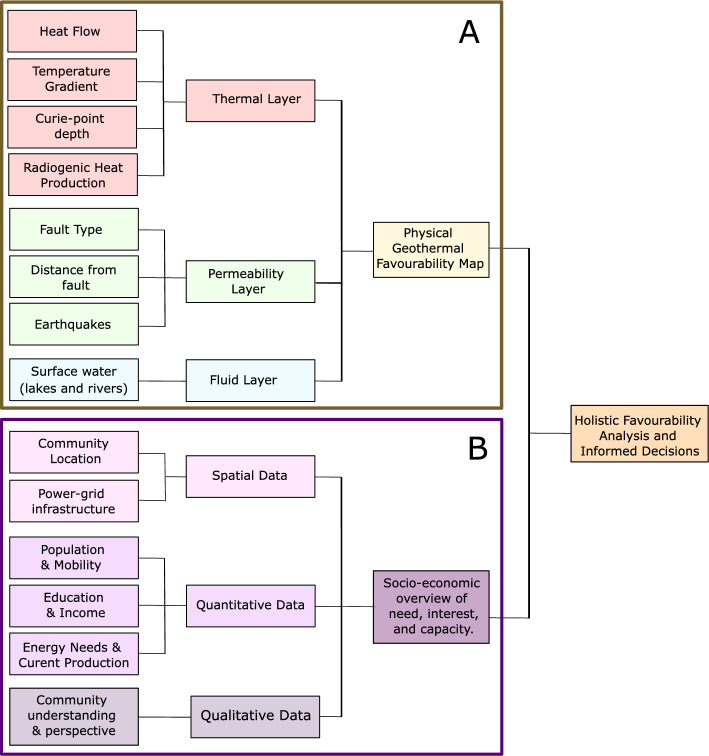
Physical and social parameters used in the geothermal favourability assessment of southwestern Yukon. The physical parameters used are shown in section A: thermal properties (red), permeability (green), and fluid availability (blue). The parameters used in section A will vary based on regional data availability. The socio-economic parameters are shown in section B and are divided into spatial data (light purple), quantitative data (purple), and qualitative data (grey-purple)

Data were sourced from the Yukon Geological Survey (2024) database and literature. The data available and used for southwestern Yukon (Table 5) are a subset of the potential data listed in Tables 1, 2, and 3. Data are grouped by category and the extent and quality of the data are described. These parameters are then incorporated into the framework presented in Fig. 2 to identify areas of geothermal favourability in southwestern Yukon.

**Table 5:** The thermal, permeability, and fluid layers that were available and used in the Play fairway analysis for southwestern Yukon

Category	Data	Data quality	Data format	Data range
Thermal	Temperature gradient	*	Point data (.csv)	12 to 60 °C km^−1^
Thermal	Heat flow	*	Point data (.csv)	54 to 126 mW m^−2^
Thermal	Curie-point depth	***	Raster file	10 to 17 km
Thermal	Qt. intrusive rocks/radiogenic heat production	**	Point data (.csv)	0.1 to 8.8 µW m^−3^
Permeability	Faults (age/length) and/or Fault system	**	Shapefile	Mapped
Permeability	Seismicity (earthquakes; post-1980)	***	Point data (.csv)	2.5 to 6.3 M
Fluid	Surface waters	**	Shapefile	Presence/absence

The quality of available data is represented by asterisks: *sporadic measurements (poor), **representative measurements, and ***interpolated over southwestern Yukon (extensive)

#### Thermal parameters

Thermal gradient has been measured in boreholes and wells (Yukon Geological Survey 2024). The majority of the thermal gradient data was retrieved from exploration or municipal wells. Thermal gradient data are available from 12 boreholes (> 100 m; 264 ± 151 m) and 10 wells (< 100 m; 47 ± 23 m). Based on the limited data available, the average (± standard deviation) thermal gradient in southwestern Yukon is 28.9 (± 15.1) °C km^−1^ (Table 5). The temperature gradient dataset is not representative of the variation in temperature gradients across southwestern Yukon as the majority (64%) of these measurements surround Whitehorse.

Heat flow should be used as a primary indicator in areas with high data density. The average (± standard deviation) heat flow in southwestern Yukon is 63.5 (± 2.8) mW m^−2^ (Davies, 2013; Yukon Geological Survey 2024). The average heat flow is based on three boreholes with heat flow data, which highlight the challenge of working in a data-scarce region.

The Curie-point depth is the depth at which a mineral or a rock loses its ferromagnetic properties (Beardsmore and Cull 2001). Magnetite is the dominant control of magnetism in crustal rocks and Curie-point depth is assumed to represent a temperature of 580 °C (Curie temperature of magnetite; Tselentis 1991). In general, the Curie-point depth is inversely proportional to the heat flow (Li et al. 2017; Witter et al. 2018). Li et al. (2017) mapped global Curie-point distribution using the global Curie-point model from magnetic anomaly inversion. The Global Curie-point model provides extensive spatial coverage globally and is therefore a useful parameter for data-scarce regions with limited on-site thermal measurements. Based on Global Curie-point model, the Curie-point depth is shallower than 14 km along and south of the Denali fault in southwestern Yukon.

The decay of radioactive isotopes is positively correlated with heat flow (Lewis et al. 2003; McLaren et al. 1999). Radioactive isotopes are common in granitic plutons. The average potential radiogenic heat production from granitoid plutons in Yukon is 2.64 ± 8.59 µW m^−3^ and the median is 1.6 µW m^−3^ (Colpron 2019). The majority of radiogenic plutons (93%) are considered low to moderate heat production (less than 6.0 µW m^−3^). Regions with moderate to high heat generation, based on granitoid pluton measurements, are within 60 km of: Haines Junction (6.4 to 7.1 µW m^−3^), Carmacks (8.8 µW m^−3^), Dawson Range (up to 7.4 µW m^−3^), and Burwash Landing (4.5 to 5.3 µW m^−3^; Colpron 2019). Previous Play fairway analyses have used proximity to Quaternary intrusions as a thermal layer due to associated remnant heat but have not considered radiogenic heat production directly despite the correlation between radiogenic heat production and heat flow (Forson et al. 2015; Siler et al. 2017).

#### Permeability parameters

Faulds and Hinz (2015) ranked eight favourable structural settings for geothermal resources. Due to data availability, these were simplified to fault types (normal, strike-slip, reverse, and unknown) for the Play fairway analysis herein. Normal faults, notably step-overs, relay ramps, terminations, and intersections in normal fault zones, are the most favourable systems. Strike-slip faults, notably intersections between normal and strike-slip faults, as well as pull-aparts in strike-slip zones were identified as less favourable than most normal fault zones. Reverse and thrust faults are ranked as lowest favourability structural settings by Lindsey et al. (2021) and are not addressed by Faulds and Hinz (2015). Faults are expected to have the greatest influence on permeability locally (Yukon Geological Survey 2024). This is represented by the assigned buffer which considers distance from fault at smaller intervals and to further distance (2 km) than the fault type layer.

Seismicity contributes to permeability by creating and maintaining open fractures. Earthquake data have been used in previous geothermal Play fairway analysis to varying extent. Lindsey et al. (2021) claimed earthquakes shallower than 20 km and greater than 2.5 M can aid in maintaining preferential flow pathways up to 5 km from epicentre. The earthquake data in southwestern Yukon was filtered to include earthquakes with a hypocenter shallower than 20 km and greater than 2.5 M (Lindsey et al. 2021; Natural Resources Canada 2021). A 5 km buffer was assigned to each earthquake (Lindsey et al. 2021).

#### Fluid availability parameters

A water table elevation or depth map would be the ideal indicator of fluid availability. However, due to the limited number of groundwater wells in southwestern Yukon, it is not possible to interpolate a water table elevation map. Other indicators used in previous studies are point measurements which, if numerous, can be used to identify areas of increased fluid availability. In southwestern Yukon, these points are sporadic and do not bring much value to the fluid layer. Here, surface fresh waters (i.e. lakes and rivers) are assumed to indicate the presence of a shallow subsurface water reservoir (Natural Resources Canada, 2017). Surface waters are the only parameter used as a groundwater indicator in southwestern Yukon. This method is most comparable to the drainage density used by Holmes and Fournier (2022).

### Parameter reclassification, weighting and superposition

**Fig. 3:**
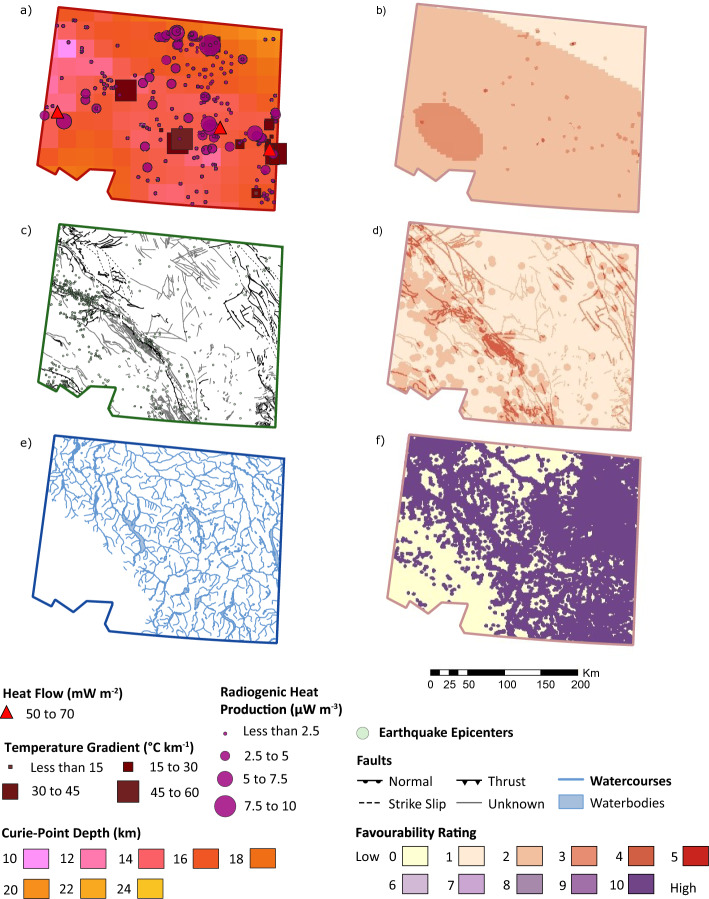
Raw data and favourability outputs generated by the Play fairway approach by layering different categories, **a**, **b** thermal layers, **c**, **d** permeability layers, and e–f fluid layer. **b** The thermal layer consists of temperature gradients (20%), radiogenic heat production (25%), heat flow (20%), and Curie-point depth (35%). **d** The permeability layer consists of fault type (33%), distance from fault (34%), and proximity to recent earthquakes (33%). **f** The fluid layer is based solely on proximity to surface water

The thermal, permeability, and fluid parameter descriptions are used in the knowledge-based approach to reclassify and assign weights to each parameter. The reclassified values are presented in Tables 6, 7, and 8, for thermal properties, permeability, and fluid availability, respectively. Parameters listed in Tables 6, 7 and 8 are equivalent to the individual parameters listed in Table 5, except the fault parameter (Table 7), which is divided into two layers to consider two aspects of the parameter: distance from fault and type of fault. The raw parameters and the superposed reclassified parameters are presented by category in Fig. 3. The fluid layer is limited to the proximity to surface water bodies. Details regarding each layer are presented in results steps 1 to 3.

**Table 6:** Reclassification for thermal properties available for southwestern Yukon

Temperature gradient (⁰C km^−1^) • 2.5 km buffer around point	Weight (20%)
Less than 15	0
15–30	2
30–45	5
45–60	8
Greater than 60	10

Radiogenic heat production A (μW m^−3^) • 2.5 km buffer around point	Weight (25%)
Less than 2.5	0
2.5 to 5.0	2
5.0 to 7.5	5
7.5 to 10.0	8
Greater than 10.0	10

Measured heat flow (mW m^−2^) • 2.5 km buffer around point	Weight (20%)
Less than 50	0
50–70	2
70–90	5
90–110	8
Greater than 110	10

Curie-point depth (km) • Interpolated over all SW Yukon	Weight (35%)
Deeper than 20	0
15–20	4
10–15	7
Shallower than 10	10

**Table 7:** Reclassification for permeability properties available for southwestern Yukon

Distance from fault (km)	Weight (33%)
Greater than 2	2
2 to 1	4
1 to 0.5	5
0.5 to 0.1	8
Less than 0.1	10

Fault type • 1 km buffer zone	Weight (34%)
Reverse, thrust, or unknown	3
Strike-slip	8
Normal and oblique-normal	10

Seismicity (Earthquakes—post-1980) • Greater than 2.5 M and shallower than 20 km • 5 km buffer zone	Weight (33%)
Absence	0
Presence	10

**Table 8:** Reclassification for fluid availability properties available for southwestern Yukon

Proximity to surface water • 2.5 km buffer zone	Weight (100%)
Absence	0
Presence	10

### Favourability maps

**Fig. 4:**
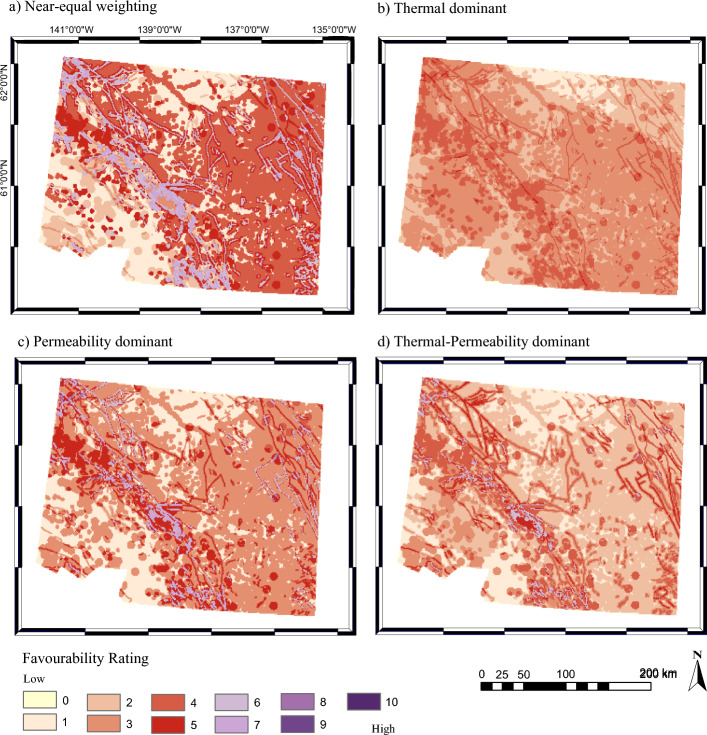
Favourability outputs generated by the Play fairway approach by layering thermal, permeability, and fluid layers (Fig. 3). The weight for each combined analysis is presented: **a** near-equal weighting between layers (34% thermal, 33% permeability, 33% fluid); **b** thermal-dominant weighting (50% thermal, 30% permeability, 20% fluid); **c** permeability-dominant weighting (30% thermal, 50% permeability, 20% fluid); **d** thermal-permeability equal weighting (45% thermal, 45% permeability, 10% fluid)

Four favourability maps were produced for southwestern Yukon by varying the weight of each category (step 7; Table 9; Fig. 4): near-equal weighting between layers, thermal-dominant weighting, permeability-dominant weighting, thermal-permeability equal weighting. The highest favourability value is 8 and only occurs in the permeability-dominant map where normal faults overlap with earthquakes. The earthquakes notably occur at normal fault terminations. The permeability-dominant layer also has the greatest distribution of areas with a favourability value of 7, notably along the Denali fault. The same highly favourable areas are visible on the thermal dominant map but with a lower absolute favourability value. Normal fault terminations with recent earthquakes remain the most favourable areas identified despite the emphasis on the thermal layer. The areas of interest are most distinct in the thermal-permeability dominant map due to the minimal contribution of the fluid layer. The thermal-permeability dominant map is the primary reference from which conclusions are drawn.

**Table 9:** Relative weight of each category used for sensitivity analysis

Weight	Near-equal	Thermal-dominant	Permeability-dominant	Equal thermal-permeability
Thermal (%)	34	50	30	45
Permeability (%)	33	30	50	45
Fluid availability (%)	33	20	20	10

### Socio-economic considerations

#### Spatial socio-economic considerations

**Fig. 5:**
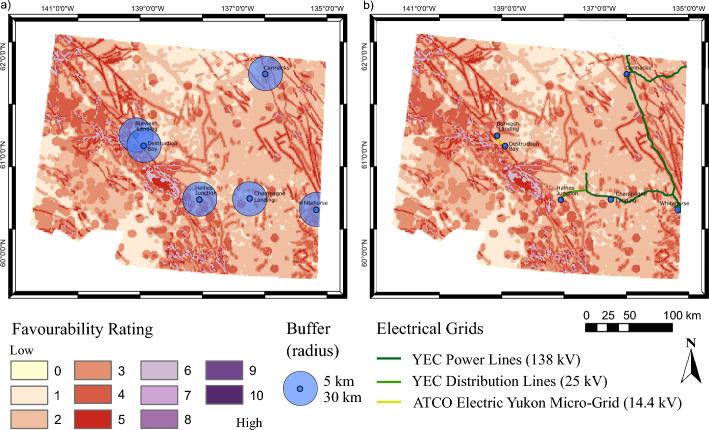
Spatial socio-economic data overlayed on the thermal-permeability dominant favourability map (Fig. 4d): a locations of communities overlayed with a 5- and a 30-km buffer, and b locations of communities and power networks including Yukon Energy Corporation (YEC) power lines, YEC power distribution lines, and low-voltage lines serviced by ATCO Electric Yukon

In Yukon, all communities rely, at least in part, on diesel for heating. Geothermal resources could offer a green-alternative to offset diesel for space-heating in any community. Within the southwestern Yukon boundaries, these communities include: Carmacks, Champagne, Haines Junction, Whitehorse, Burwash Landing and Destruction Bay (Fig. 5a).

Carmacks, Burwash Landing and Destruction Bay are the communities in southwestern Yukon located within physically favourable regions for geothermal development (Fig. 5a). A highly favourable section is located between Destruction Bay and Haines Junction, but there is no community nearby to benefit from any potential resource development in the area.

Unlike other southwest Yukon communities, Burwash Landing and Destruction Bay are not connected to the Yukon electrical grid and are instead serviced by a micro-grid comprising thermal (diesel) generation and low voltage transmission (Moorhouse et al. 2020; Fig. 5b). Geothermal research would have the greatest impact on these communities because the current low voltage transmission and thermal generation does not allow for electrical space heating in either community. Here, we present the evaluation of the socio-economic context of Burwash Landing and Destruction Bay as an example of the integration of socio-economic analysis into the Play fairway approach.

Burwash Landing and Destruction Bay are located within the traditional territory of Kluane First Nation (KFN), one of 11 self-governing First Nations in Yukon who operate in tripartite with Yukon Government and Canada. Burwash Landing and Destruction Bay are on the southwest of Łù'àn Män (Kluane Lake), less than 20 km apart. The seat of the Kluane First Nation Government is in the community of Burwash Landing, where most Kluane First Nation citizens also reside. Historically, Yukon Government services such as the school, highway camp, and nursing station have been located in the smaller community of Destruction Bay. As Burwash Landing grows, it is expected that more services will be relocated to that community.

#### Quantitative socio-economic results

Statistics from the Canadian Census and the Canadian Energy Association can provide insight into population dynamics from 2006 to 2021 and current energy needs. Supplemental information is available from the Canadian Energy Agency and other territorial reports. This quantitative information provides a baseline reference of the socio-economic status to assess need, interest, and community capacity prior to potential development (Chitsaz, 2022).

The population of Burwash Landing is greater and more stable than the population of Destruction Bay (Table 10). The population increased from 2006 to 2011 but decreased from 2011 to 2021 (Statistics Canada, 2012, 2023). The population of Burwash Landing is 13% lower in 2021 relative to 2006, whereas the population of Destruction Bay varied by up to 44% with an average decrease of 32% between 2006 and 2021 (Statistics Canada, 2012, 2023).

**Table 10:** Population variations over the past 15 years (Canada Census; Statistics Canada, 2007, 2012, 2017, 2023)

Population and mobility	2006	2011	2016	2021
Burwash landing				
Population	73	95	72	64
Occupied private dwellings	41	50	47	44
Median age	45.5	41.5	50.7	47.2
* % moved within 5 years*				
* Intraprovincial migrants*	–	–	20	15
* Interprovincial migrants*	–	–	10	10
Destruction bay				
Population	55	35	55	40
Occupied private dwellings	24	17	22	16
Median age	48.2	–	50.5	54.8
% moved within 5 years				
* Intraprovincial migrants*	–	–	0	–
* Interprovincial migrants*	–	–	10	–

“-” marks values not available from the Census. The census refers to intraprovincial and interprovincial migrants, note that intraprovincial refers to intraterritorial for Yukon territory, and interprovincial migrants refers to migrants who have moved from another territory or province

Table 11 identifies available skills within the community based on the Canadian Census (Statistics Canada 2023). In Burwash Landing, the difference between the labour force participation rate and the employment rate is 16.7%. The majority of Burwash Landing community members (over 15 years old) have completed secondary education (75%) and many (58%) have obtained a postsecondary certificate, diploma, or degree. In contrast, all Destruction Bay residents (over 15 years old) have completed secondary education and the majority (75%) have obtained a postsecondary certificate, diploma, or degree. The difference between the labour force participation rate and employment rate (12.5%) is comparable to Burwash Landing.

**Table 11:** Education and skills in Burwash Landing and Destruction Bay (Canada Census; Statistics Canada 2023)

Education and labour force	Burwash Landing (2021)	Destruction Bay (2016)
*Highest education attainment*	Sample size: 60	Sample size: 40
No certification, diploma, or degree (%)	25	0
Secondary (%)	17	25
Postsecondary certificate, diploma, or degree (%)	58	75
*Labour force*	Sample size: 60	Sample size: 40
Labour force participation rate (%)	75	75
Employment rate (%)	58.3	62.5
Unemployment rate (%)	22.2	33.3

Burwash Landing and Destruction Bay are connected by a micro-grid. The main power supply for both communities are the three diesel generators located at the power plan in Destruction Bay. The power plant is able to meet 100% of the electricity demand for both communities (Table 12). However, Kluane First Nation has prioritized renewable energy and initiated clean energy initiatives including the installation of solar panels and the development of a micro-grid connected wind turbine and battery energy storage system (Table 12; 2023). Based on an energy census by the Council of Yukon First Nation (2023), solar energy currently represents 20% of electricity used by the Kluane First Nation Government (288 GJ). The remainder is from the grid-provided diesel generation (80%; 1 123 GJ).

**Table 12:** Combined energy usage for Burwash Landing and Destruction Bay (CYFN, 2023)

Community	Input energy	Production (%)	Production (GJ)
Burwash landing and destruction bay	Electricity	19	6907
	Heating oil	22	7998
	Propane	2	727
	Wood	26	9452
	Gasoline	29	10,543
	Diesel	3	1091
	Total	100	36 354

#### Qualitative socio-economic results

##### Community openness to development

Kluane First Nation is actively engaged in community development and the transition to clean energy as demonstrated by existing solar and wind developments. These initiatives decrease current diesel demand and increase total available energy to support future developments.

##### Community goals and projections

Kluane First Nation is currently in the process of building a new school in Burwash Landing to replace the current school in Destruction Bay. The Kêts’ádań Kų̀ (House of Learning) aims to retain families in the community by expanding on community-based elementary and secondary education. Retaining families would increase population size, decrease the median age, and increase the number of individuals actively seeking employment. This expected increase in population is reflected by Kluane First Nation’s bid for contractors to build 4 new 5-bedroom detached homes in Burwash Landing. The commissioning of the wind project in 2024 will temporarily decrease CO_2_ emissions from 1309 to 700 tCO2e, but emissions are expected to return to comparable levels (1290 tCO_2_e) by 2050 assuming no further development of renewable resources (Council of Yukon First Nation, CYFN, 2023). The increase in energy demand will be due to population growth, infrastructure expansion, and a transition to electric cars (CYFN, 2023).

## Discussion

Play fairway analysis is a useful tool to identify areas of resource favourability and has been successfully used in geothermal exploration in recent years (e.g. Ito et al. 2017; Lindsey et al. 2021; Siler et al. 2017; Wang et al. 2021). However, Play fairway analyses are typically only applied to data dense areas and are limited to physical parameters. A holistic approach to evaluate local geothermal potential in remote regions should include discussing the limitations of Play fairway analysis due to data scarcity and incorporating socio-economic parameters in the resource assessment. This will ensure that target communities’ best interest is considered as of the exploration stage.

In areas with high data density, direct point measurements of heat, permeability, or groundwater level can be used to create maps with significant coverage by interpolation. In areas with low data density, these data points are sporadic and cannot be reliably interpolated over large areas. For example, in southwestern Yukon (⁓ 250 by 310 km), only three heat flux measurements are available (Davies, 2013; Yukon Geological Survey 2024). Therefore, proxy data must be used to evaluate thermal properties, permeability, and groundwater availability on a regional scale. For southwestern Yukon, significant proxy parameters for the thermal layer include Curie-point depth and radiogenic heat production. Curie-point depth has been mapped globally (Li et al. 2017) and extensive coverage is therefore available in most study areas but has not been used in previous geothermal Play Fairway analysis (Table 1). The radiogenic heat production was recently mapped for Yukon (Colpron 2019) but may not be as well known in other regions. Potential parameters for each category are presented in Tables 1, 2, and 3, but data availability controls the weight assigned to each parameter. The direct and indirect parameters provided herein can be used irrespective of the geothermal play-type.

Through a Play fairway analysis in areas with low data density, a favourability map is developed. This differs from a probability map which can be used to estimate the potential quantitatively. A favourability map identifies which areas should be pursued locally and aims to provide insight into the information currently available. The framework presented herein provides insight into potential parameters and the approach to scale and weight parameters. However, the scale of each parameter must consider the local context and should not only be compared to global values. For example, Siler et al. (2017) considered heat flow in the geothermal Play fairway analysis of Modoc Plateau region (⁓ 225 by 350 km), U.S.A. Across the Modoc Plateau region, heat flow ranged from 65 mW m^−2^ to 105 mW m^−2^, where 65 mW m^−2^ was considered low favourability and 105 mW m^−2^ was considered high favourability. In southwestern Yukon, the heat flow across the three-available data points is 60.0 mW m^−2^ in Whitehorse, 66.4 mW m^−2^ near Mt. Lucania, and 64.0 mW m^−2^ between Whitehorse and Haines Junction. Due to the limited and poor spatial distribution of the available data, the reclassification used was scaled around, but not limited to, the 60 to 66.4 mW m^−2^ range. This is to ensure the Play fairway analysis could be easily updated should more data points become available. The current heat flow data available for southwestern Yukon, less than one measurement per 25 000 km^2^, is insufficient to represent regional heat flow variability.

Using multiple data sources within each category can provide a more extensive spatial coverage, but parameters within the same category may not always be positively correlated. For example, in southwestern Yukon, areas with the shallowest Curie-point depth are not areas with the greatest radiogenic heat production despite both being indicators of potentially elevated heat flux. This discrepancy is because: (1) radiogenic heat production is based on surface granitoid measurements and does not consider buried plutons; (2) the radiogenic heat production presented as point data and not adjusted to pluton size, and (3) Curie-point depth is also influenced by local heat flux and tectonics. Both parameters provide insight into local heat potential and can be complementary but are not mutually exclusive. This also applies to permeability parameters.

Elevated permeability is expected within a fault damage zone due to increased fracture density. Fault and fracture permeability depend on fault type and age (Ito et al. 2017; Lindsey et al. 2021; Siler et al. 2017; Wang et al. 2021). Fractures close over time, but can be maintained by seismicity. Previous research in data-rich areas use varying filters to consider the influence of earthquakes on maintaining local permeability. For southwestern Yukon, the earthquake data were filtered based on Lindsey et al. (2021) approach: earthquakes shallower than 20 km and greater than 2.5 M can aid in maintaining preferential flow pathways up to 5 km from epicentre. However, in an area with extensive shallow earthquakes, the filter suggested by Wang et al. (2021) or Ito et al. (2017) may be more appropriate (described in supplementary material). Ito et al. (2017) limited the seismic data to earthquakes shallower than 5 km (⁓40 000 events). Wang et al. (2021) concentrated on micro-earthquakes < 3 M and shallower than 4 km.

In southwestern Yukon, earthquake epicentres typically occur along or at the end of faults. For example, seismicity at normal fault terminations around Carmack’s can contribute to maintaining local permeability in that area. However, not all seismicity is recorded in highly fractured surface areas. There are few faults mapped at the surface south of Koidern next to the Alaskan border, yet seismicity is high. The earthquake data are plotted at the surface based on locations of epicentres. However, the epicentre of a low-angle fault (such as a thrust fault) may be far from the surface trace of the fault. It is important to pay particular attention to earthquakes located on or near surface-trace faults because both the earthquakes and surface faults and fractures are required to maintain permeability. When assigning weights and analysing results, it is essential to understand the interactions between each parameter and their context.

Hot springs have been used in the heat, permeability, and fluid layers of previous geothermal Play fairway analyses (Forson et al. 2015; Holmes & Fournier 2022; Lindsey et al. 2021; Wang et al. 2021). However, mapped hot and warm springs were excluded from the geothermal Play fairway analysis of southwestern Yukon. It is essential to ensure that the parameters selected are available but are also pertinent based on the geological setting. Hot and warm springs were excluded from the heat category because they have not been correlated with elevated heat flow in southwestern Yukon (Witter and Miller 2017), which is common to hot springs of the Canadian Rockies (Ferguson et al. 2009). The Takhini hot springs temperature profile suggests that convection is the dominant heat transfer mechanism responsible for this thermal manifestation. Deep groundwater is brought to the surface by permeable layers within inclined sedimentary rocks facing the regional groundwater flow direction (Langevin et al. 2020; Léveillée-Dallaire and Raymond 2023). In an orogenic setting such as southwestern Yukon, the presence of hot springs is therefore not a strong indicator of the geothermal potential. The mapped hot springs were also not included in the permeability layer as they overlap with faults, which have a greater control on fluid flow.

The geothermal exploration borehole recently drilled in the Takhini Hot Springs area (Fraser et al. 2019) can, however, help to validate the reliability of our Play fairway analysis. The rating revealed by the Play fairway analysis around the Takhini hot springs is low in all renditions of the favourability maps. Witter et al. (2018) previously estimated heat flux in the Takhini hot springs area at 50 mW m^−2^. The low heat flux estimated is in line with the site-specific study by Langevin et al. (2020), which presents a conductive temperature gradient of 16 °C km^−1^ based on temperature data recorded from 50 to 450 m. Léveillée-Dallaire and Raymond (2023) then used hydrothermal modelling to understand the origin of the hot springs. The presence of the hot springs was determined to be associated with inclined fractured sedimentary layers that created a preferential flow pathway for deep groundwater to reach the surface, resulting in the hot springs (Léveillée-Dallaire and Raymond 2023). The hot springs are currently exploited for tourism, but the geothermal potential at-large is deemed low due to the low terrestrial heat flux and unit specific permeability. This is accurately portrayed by the Play fairway analysis presented herein, partly validating the proposed physical method. Fluid availability is not expected to be a limiting factor in southwestern Yukon.

Previous studies in regions where groundwater availability at some depth is expected either excluded the fluid layer from the Play fairway analysis or assign a low weight to the fluid layer (Lindsey et al. 2021; Olvera-García et al. 2023; Siler et al. 2017; Wang et al. 2021). Here, surface fresh waters (i.e. lakes and rivers) are used as a proxy for fluid availability at depth and provide insight into groundwater distribution on a regional scale. The use of fresh surface waters is unique to this study and should only be used where groundwater table measurements are sparse. Ice cover is not included in the surface waters layers as it is not associated with water table depth. However, ice cover melt rate could be considered in combination with recharge rate in areas where ice cover melt significantly contributes to groundwater recharge.

Alternative non-surface groundwater indicators include hydrothermal alteration (Lindsey et al. 2021), water table elevation, groundwater recharge, and electrical resistivity (Ito et al. 2017). Electrical resistivity measurements are available for small areas within southwestern Yukon, such as the Duke River area but are not sufficient to contribute substantially to a regional Play fairway analysis. They could be used to support interest if electrical resistivity data are available for areas identified from the regional analysis.

The main limitation in using Play fairway analysis to identify areas of physical geothermal favourability in a remote area is data availability. This limitation is reflected in the favourability maps for southwestern Yukon (Fig. 4). The physical methodology presented here closely resembles that of Lindsey et al. (2021) to identify areas of geothermal favourability in north-western Argentina. Both studies categorize parameters into thermal, permeability, and fluid availability using a knowledge-driven approach to define the favourability of each parameter within the categories. Lindsey et al. (2021) then compared favourability maps using the sum and product model. The sum model is identified as being the optimal solution for data-scarce regions and is therefore presented herein.

A sum approach is used to include all data layers that are represented at any point, but a value of 0 is assigned to each parameter when that parameter is not present (Lindsey et al. 2021). This results in the low favourability values. This method is unable to distinguish between an area with low-favourability due to data scarcity or data associated with an environment which is not conducive to geothermal resources. It is therefore essential to use a sensitivity analysis as well as compare favourability maps with a data completeness map to understand why an area is discarded. For southwestern Yukon, the same areas of interest were highlighted irrespective of the weighting of each layer. However, should an area be more prominent on a permeability-dominant map compared to a heat- or equal-weight map, this may be due to a lack of heat data availability rather than low heat potential. Category weights cannot be mixed within a map to avoid bias. However, conducting a sensitivity analysis and comparing various weights can help identify areas with high favourability dominated by one category which could be used to focus future data collection efforts.

A Play fairway analysis in data-scarce regions is a useful tool to identify preliminary areas of interest. In southwestern Yukon, the areas of interest for further exploration based on physical parameters are along the Denali fault primarily due to a shallow Curie-point depth and high fault density, and near Carmacks due to normal fault terminations and associated seismicity.

Previous Play fairway analyses concentrated on physical parameters only, but geothermal energy is a local resource that cannot be easily exported. A holistic approach to evaluate local geothermal potential in remote regions should include discussing the limitations of Play fairway analysis due to data scarcity and incorporating socio-economic parameters in the resource assessment. This approach will ensure that target communities’ best interest is considered at the exploration stage. To complete a holistic evaluation of local geothermal potential in remote regions, the physical analysis must be complemented with a socio-economic analysis of targeted sites. By identifying communities using spatial, quantitative, and qualitative socio-economic data researchers can ensure that research and development occur in the best interest of the intended user. In remote regions, the success of a project depends on community capacity, investment, and collaboration (Stefanelli et al. 2019). Beckley et al. (2008) define community capacity by the ability of a community to combine social, economic, natural, and human capital to reach desired outcomes. The Canadian Census (Statistics Canada, 2007, 2012, 2017, 2023) and Council of Yukon First Nation (2023) data provide baseline data with regard to local community capacity but must be complemented by qualitative data to see the full picture.

Once a project is deemed physically feasible and socially sustainable, further analysis should consider the economic viability. The economic viability depends on the current cost of energy in the community of interest and the type of geothermal technology considered (Majorowicz and Grasby 2014). Miranda et al. (2022) discuss the techno-economic feasibility of four different types of geothermal technologies in northern Canada: Ground-coupled heat pumps (GCHP), borehole thermal energy storage (BTES), enhanced geothermal systems, and hybrid geothermal systems. GCHPs, notably solar-assisted GCHPs, are expected to be technologically and economically viable in northern regions and are a relatively low-cost and low-risk applications for remote communities to integrate geothermal energy (Gunawan et al. 2020; Moreno et al. 2022). In contrast, enhanced geothermal systems could be used to produce electricity in remote regions but present a high technological risk and would require significant investment (Miranda et al. 2021). Alternatively, current BTES research explores the potential for seasonal storage and recovery of waste heat from diesel generators, which could be useful to heat remote communities that remain reliant on diesel for electricity (Ghoreishi-Madiseh et al. 2019). Important heat loses are expected underground such that BTES are best suited for very-low temperature heating applications requiring a heat pump (Giordano and Raymond 2019). BTES carries moderate technological risk and would require a moderate investment, depending on the depth of boreholes required; reusing pre-existing boreholes (exploration, hydrocarbon wells) would significantly reduce the financial investment (Gascuel et al. 2022). The socio-economic analysis should aim to understand the current energy context, identify local stakeholders, and evaluate the communities’ willingness to assume technological and economic risks, such that an appropriate range of options can be presented to the community.

Through the holistic analysis for geothermal favourability in southwestern Yukon, the communities of Burwash Landing and Destruction Bay were identified based on their energy needs and the Kluane First Nation Government’s support for renewable energy projects and community development. The spatial data allowed us to identify these communities based on their proximity to physically favourable environments for geothermal exploration. The quantitative data provided insight into the community population and energy needs, but it is essential to consider qualitative data in the socio-economic analysis. Based solely on the quantitative data, population fluctuates in both Burwash Landing and Destruction Bay from 2011 and 2021 (Statistics Canada, 2012, 2023). There is no clear population trend for such small communities. However, when complemented with the qualitative data, we conclude that the population and energy demands are expected to increase with new developments underway in Burwash Landing. Coupling quantitative and qualitative data is essential in remote communities where population size is small, and trends are variable.

The primary energy demand in Burwash Landing and Destruction Bay is for space-heating. Currently, remote communities in Yukon are not permitted to connect their district heating to the electrical grid. Buildings in these communities are heated by oil or biomass. The energy limitations influence energy needs in communities. Understanding these limitations can better inform the type of energy development that would benefit the community. Electricity can be shared between Burwash Landing and Destruction Bay due to the micro-grid, but they are too far apart for both communities to benefit from geothermal energy for space-heating from the same source. As the space-heating demand is greatest, and growing, in Burwash Landing, future research should evaluate the potential for electricity production and space-heating within 5 km of the community. Space heating could be considered either as the sole use of shallower resources should electricity production be deemed not viable, or as a secondary usage of waste heat from electricity production.

The qualitative data used herein are based on announcements and reports by the Kluane First Nation Government. This provides context for the quantitative data but is not a form of direct communication with community members. We propose that direct community consultation should be the next step to understand community member’s perspective on transition to renewable energies and the current social understanding and acceptability of geothermal energy. Community surveys and interviews provide an opportunity to connect with community members and better understand community needs and interests.

The support of the local community is essential for the continuation of research and any potential geothermal development in the region. By coupling the quantitative and qualitative results, we were able to evaluate the socio-economic context around Burwash Landing and Destruction Bay which better informs decisions to pursue further geothermal exploration and potential development.

## Conclusions

This research highlights the importance of using a holistic approach to evaluate the low-temperature geothermal favourability in remote regions based on physical and socio-economic parameters. The framework provided herein aims to facilitate future analyses of geothermal potential in remote regions by identifying proxy parameters to evaluate heat, permeability, and fluid availability as applicable to geothermal systems.

Proxy data with large spatial coverage need to be used in favourability analyses for remote and data-scarce regions where direct data are unavailable. For example, in southwestern Yukon, the most reliable indicators of thermal potential, such as temperature gradient and heat flow data, are too scarce to be useful. The Curie-point is therefore used as a first-order approximation of relative thermal potential within the region although it is not a direct indicator of temperature. This is a common limitation of proxy data with large spatial coverage. Proxy data used in previous analyses and additional parameters applied to the Play fairway analysis of southwestern Yukon are presented in Table 5. These parameters can be integrated into Play fairway analyses in data-scarce regions where in situ measurements are limited but should be considered with care. The significance of any parameter applied to a Play fairway analysis will depend on the local context, data availability, and spatial coverage. Due to the limitations of data-scarce regions, it is essential to recognize that the combination of the physical parameters (heat, permeability, and fluid) results only in favourability maps. In areas with extensive data coverage, a probability map can be produced using Play fairway analysis. A probability map can be used to quantify the geothermal potential, whereas a favourability map can be used to highlight areas of interest based on available data and highlight where further data collection may be required to make informed decisions. For southwestern Yukon, additional exploration boreholes would decrease uncertainty by providing direct temperature measurements and information required to calculate local heat flux.

The framework presented herein also demonstrates the importance of considering both physical and socio-economic parameters when evaluating geothermal favourability. It ensures that further research in areas identified as favourable for geothermal exploration and development considers the needs, limitations, and desires of remote communities. Sustainable and community-centric geothermal projects can be pursued effectively by using a holistic approach in the exploration stage.

The holistic Play fairway analysis presented herein, identifies Burwash Landing and Destruction Bay as areas for further research within southwestern Yukon. Community engagement is encouraged in the communities of Burwash Landing and Destruction Bay to understand the social acceptability of geothermal energy, and the energy demand that geothermal energy could be used to address. A strong understanding of community needs and interest should be used to guide the next steps in the evaluation of geothermal potential based on the community’s needs and systems of interest. Further socio-economic analysis is encouraged to understand the energy demand in other regions of elevated physical favourability in southwestern Yukon, including Carmacks and Haines Junction.

## Supplementary Information

Supplementary material 1.

## Data Availability

The datasets analysed during the current study are available from GeoYukon and Natural Resources Canada repositories: [https://map-data.service.yukon.ca/geoyukon/Geological]. [http://earthquakescanada.nrcan.gc.ca/stndon/NEDB-BNDS/bulletin-en.php]. [https://open.canada.ca/data/en/dataset/9d96e8c9-22fe-4ad2-b5e8-94a6991b744b]. The relevant references have been cited for any data from publications which are not included in the references listed above.
