# Supplementary material for: Geothermal favourability in data-scarce regions: incorporating physical and socio-economic factors into a modified Play fairway approach, southwestern Yukon, Canada
Source: Geotherm Energy (Heidelb). 2025 May 16;13(1):24. doi: 10.1186/s40517-025-00345-6 (PMC12084253; doi:10.1186/s40517-025-00345-6)
Supplement: Supplementary file 1 — Supplementary material 1. [file 40517_2025_345_MOESM1_ESM.docx]

**Thermal Properties**

Appendices A, B, and C provide an explanation for all potential parameters that could be used to generate heat, permeability, and fluid layers, respectively. Parameters identified by an asterisk (*) are used in the example of southwestern Yukon. Examples of how different parameters were used and weighted in other Play Fairway analyses are provided for reference. Note that these categorization are unique to their respective study areas and should be adjusted based on local context.

***Heat Flow –** Heat flow measurements are direct measurements from boreholes where temperature gradient and thermal conductivity have been measured. Heat flow is the most reliable parameter in the thermal layer as it is a direct measurement which typically does not vary within the first kilometers of the Earth crust.

Heat flow maps can be generated where measurements are abundant, or point-measurements can be used. In areas with simple geology and abundant heat flow measurements, additional heat flow values may be estimated from thermal gradients and a linear regression of temperature gradients and heat flow in the study area (Siler et al., 2017). Alternatively, if the stratigraphy is known, literature values of thermal conductivity could be used to improve heat flow database (Holmes & Fournier, 2022). However, prior to estimating the heat flow based on the temperature gradient, it is essential to ensure that the temperature gradient is controlled by a conductive system. Siler et al. (2017) identified some shallow wells (< 30 m) with elevated temperature gradients (⁓ 400 ⁰C/km). These temperature gradients were representative of a convective system and could not be used to evaluate heat flow.

Example of Heat Flow Classification Scales: Heat flow values are bound based on regional high and low values. For example, Siler et al. (2017) defines a low to high favourability range of 65 to 105 mW/m^2^ as per the regional heat flow measurements.

***Temperature Gradient** – Temperature gradient is the most common heat parameter used in geothermal Play fairway analysis. Temperature gradient measurements from individual boreholes can be used directly when it is not possible to estimate the heat flow based on the regional context.

Temperature gradient datasets can be improved by including wells with bottomhole-temperature data and estimating the gradient based on the average surface temperature (Forson et al., 2015; Nielson et al., 2015, Siler et al., 2017; Holmes et al., 2022). Siler et al (2017) assign a greater uncertainty to regions where the thermal gradient is determined based on bottomhole temperature.

With sufficient data density, Krigging interpolation can be used to estimate continuous temperature gradients across the area of interest (Forson et al., 2015). Where data density is insufficient for interpolation, each data point is considered representative of ⁓ 2×2 km cell size (Siler et al., 2017). Temperature gradients are applied to buffer of 2.5 km in the southwestern Yukon example.

An alternative to representing well temperature as temperature gradient is to use temperature at 3 km depth (Hinz et al., 2016; Faulds et al., 2018). This eliminates uncertainty from shallow well temperature gradients. The depth of wells from which temperature gradients are presented should be considered with more certainty associated with deeper wells.

**Groundwater temperature –** Ito et al. (2017) use temperature anomalies based on groundwater temperature, rather than heat flow or temperature gradient in the Play fairway analysis across the state of Hawaii. Ito et al. (2017) identify the anomalies based on groundwater temperature using the following approach:

1. Subtract mean local surface temperatures from measured water temperatures.
2. Find best fit-line to all excess temperatures versus depth and subtract from each measurement.
3. Standardize the temperature anomalies.

Ground temperature anomalies is an alternative method which quickly compare heat potential between sites. If no direct heat flow or temperature gradient measurements are available, groundwater anomalies could be used instead.

Siler et al. (2017) use groundwater temperature data to bonify the temperature gradient dataset but do not include a groundwater temperature layer directly into the Play fairway analysis.

***Curie Point Depth** - The Curie-point depth is the depth at which a mineral or a rock loses its ferromagnetic properties (Beardsmore & Cull, 2001), which is typically assumed to be at 580 ⁰C (Curie temperature of magnetite; Tselentis, 1991).

**Hot Spring Temperature –** Hot or warm springs are surface indicators of warm water. Forson et al. (2015) use proximity to springs weighted by temperature as an indicator in the heat layer. Individual springs were converted to a raster buffered to 0.8 km. Areas around individual springs were also assigned a favourability based on proximity (Euclidean distance analysis) and spring temperature. Forson et al. (2015) do not consider the origin depth of spring water.

Lindsey et al. (2021) and Wang et al. (2021) assigned favourability based on hot spring temperature. Lindsey et al. (2021) assigned a weight of 10/25 in the heat layer at the following scale: 0 = *<* 15 ⁰C; 0.2 = 15 to *<*30 ⁰C; 0.5 = 30 to *<*50 ⁰C; 0.8 = 50 to *<*70 ⁰C; 1 = *>* 70 ⁰C

In the Play fairway analysis completed by Lindsey et al. (2021) and Wang et al. (2021) direct subsurface temperature measurements were not included.

Hot spring temperature provides an alternative heat information. The origin depth of the hot spring water should be considered based on the local geological context before being used in a Play fairway analysis. For example, hot spring discharge in a Cordillera is typically heated at depth (Fergusson et al., 2009). Langevin & Raymond (2020) confirmed that the Takhini hot springs in southwestern Yukon result from deep fluid circulation being brought to the surface along faults. Hot spring data was therefore not included in the Play fairway analysis for southwestern Yukon because they are not representative of shallow thermal resources.

**Geothermometry and Geochemistry –** Geothermometry on surface springs can be used to infer temperature at depth. Studies used proximity to springs and inferred temperature from geothermometry as a heat layer parameter (Forson et al., 2015; Nielson et al., 2015). Some studies also used geothermometry to infer temperature at depth based on well water samples (Nielson et al., 2015; Faulds et al., 2018). Faulds et al. (2018) were able to infer subsurface temperatures of 130-140 ⁰C using geothermometry.

Ito et al. (2017) and Lautze et al. (2017) use Cl/Mg (ratio) and SiO_2_ (ppm) as geothermal water indicators. A high concentration of Cl and a low concentration of Mg is expected in geothermally altered seawater. Both Cl and SiO_2_ are more soluble in geothermal waters whereas Mg precipitates. Therefore, the concentrations and ratios can be used as a geothermometer. These are point values from wells or surface springs.

Siler et al. (2017) addresses SiO_2_, and K/Mg but these data are sparse. The primary geothermometer used in this study is Na/K. Siler et al. (2017) assigned ratio ranges to identify a high, medium, or low heat based on geothermometry.

Wang et al. (2021) and Holmes et al. (2022) use SiO_2_ as a geothermometer. Holmes et al. (2022) used the Fournier chalcedony geothermometer relationship to estimate reservoir temperatures based on SiO_2_ concentrations. This resulted in reservoir temperatures ranging from -28 to 138 ⁰C. Using this relationship, negative reservoir temperatures are expected, though physically unrealistic, when SiO_2_ concentrations are low (Holmes et al., 2022). Holmes et al. (2022) also use lithium and boron concentrations and highlight the importance of considering relative values, rather than absolute values, when using geothermometers in a Play fairway analysis.

**Hydrothermal alterations –** Primary minerals can be altered when in contact with geothermal fluids. Hydrothermal fluids can therefore be indicators of remnant heat. Alterations depend on the primary mineral composition, fluid composition, temperature, permeability, and pressure (Poux & O’Brien, 2020). Poux and O’Brien (2020) and Lindsey et al. (2021) include hydrothermal alterations as a heat indicator.

Poux and O’Brien (2020) identified epidote or actinolite as indicators of the highest temperature zones of the reservoir. Poux and O’Brien (2020) also use alternation minerals (such as smectite, illite, chlorite) to identify depth and thickness of a clay-cap which may seal a geothermal reservoir which contributed to the development of a 3D geological model of the study site and associated degree of geothermal favourability (Poux and O’Brien, 2020).

Lindsey et al. (2021) presents hydrothermal alteration mineral information as a gridded raster layer considering argillic or phyllic alteration classified by age indexed from older than Miocene or unknown age (0.01) to estimated age is *<* 1 Ma (1). Of the three parameters used in their heat model, hydrothermal alterations were weighted the lowest (6/25).

These indexes should be changed based on the alterations expected in the study area which will depend on the primary minerals and fluid chemistry.

**Rift Zone and Stage –** Proximity to rift zones and the rift zone stage have been used as heat indicators in Play Fairway analyses across Hawaii (Ito et al., 2017; Lautze et al., 2017). The correlation between different rift zones, volcanic activity, and geothermal resources is discussed by Hinz et al. (2016) with respect to the East African Rift System but is not used in the Play Fairway analysis.

Rift zones are included by Ito et al. (2017) and Lautze et al. (2017) because subsurface magma is known to exist under these zones. The subsurface magma contributes to increase ground temperature and the prolificacy of geothermal resources. However, Lautze et al. (2017) caution that, due to the shape, rift zones typically lose heat faster than other volcanic features such as calderas.

Ito et al. (2017) define a negative exponential distribution to identify heat probability based on distance from a rift. The maximum heat probability is 0.6 at 0 km from a rift zone. Lautze et al. (2017) then use a 1-10 ranking system to weight each component. Rift zones (7) are weighted below calderas (7) but above vents (4) and dikes (5). These values are assigned to 9 variables in the heat layer with a cumulative weight of 63. For comparison, the highest weighted variables are the well temperature (9) and magnetotelluric resistivity.

**Volcanic vents (including calderas) and/or volcanic centers** – Volcanic vents can be used in heat layers as, similar to rift zones, they are indicative of subsurface magma. Forson et al. (2015) created different size buffers around vent types: stratovolcanoes (5 mi), calderas (3 mi) and minor vents (1.5 mi). The weighted value was unique to each vent as it depended on age and rock type. The weight then decreased with distance from the buffered area.

Ito et al. (2017) define a negative exponential function to identify heat probability based on distance from a volcanic vent. The maximum heat probability is 0.45 at 0 km from volcanic vent compared to 0.6 for rifts. The probability function for the volcanic vents follows the same trend as distance from a rifts. The overall heat probability of volcanic vents is lower than rifts and are weighted less (See *Rift Zone and Stage* for relative weighting of volcanic vents in Lautze et al. (2017)).

Nielson et al. (2015) discuss the intention to include balsatic vent distribution in the Play Fairway analysis but have yet to define the heat probabilities and weight.

**Volcanism (Age/Recency & Type)** – Recent volcanism is associated with residual heat from magmatism. Rock type is considered as it is associated with the depth of original magma chambers (Lindsey et al., 2021). Siler et al. (2021) prioritise age over composition, but both are considered on a sliding scale out of 1 (Holocene: 0.8-0.9; Pleistocene: 0.5-0.7; Quaternary/Tertiary 0.2-0.4). Lindsey et al. (2021) also considers the age and type by using a normalized sum of weights assigned for weight and type. Unlike Siler et al. (2021), Lindsey et al. (2021) includes a weight for intermediate composition (0.9) and gives all volcanism under 1 Ma and age weight of 1 Ma. The buffer area over which these weights are applied are not specified.

**Uplift Rate –** Uplift rate is used by Wang et al. (2021) in a play fairway analysis of Taiwan as due to the presence of an active orogenic mountain belt (4 – 10 mm per year). Many geothermal play fairway analyses occur in areas with active or historic volcanism. However, previous research identified that heat sources in Taiwan are associated with the uplift of the Central Mountain Range rather than volcanism. The uplift rate is one of three parameters used in the heat layer with a weight of 0.25. Uplift measurements are point data and inverse distance weighting is used to interpolate uplift rates over study area.

**Dike Density –** Dikes are related recent or historic presence of magma. Sporadic dikes remain an indicator of magmatic activity, but high dike density is suggestive of larger magmatic systems such as rift zones or cordilleras (Lautze et al., 2017). These environments are expected to have higher residual heat, therefore dike density can be used as a proxy in the heat layer. Holmes et al. (2022) used units degree degree^-2^ to represent dike density which was calculated based on the available dike polyline dataset. Neither use of dike density considers the age or type of dike (Lautze et al., 2017; Holmes et al., 2022).

***Intrusive Rocks and/or Radiogenic Heat Production –** Forson et al. (2015) considered the areal surficial extents of young silicic intrusive rocks. Intrusive rocks considered were Pliocene to Holocene in age and rhyodacitic to andesitic in composition (Forson et al., 2015). Similar to dikes, intrusive rocks are indicative of past magmatic activity with potential for residual heat. Felsic intrusive rocks also contain radiogenic isotopes which contribute to local heat production as they decay (McLaren et al., 1999).

For southwestern Yukon, radiogenic heat production data was available from plutons across the study area (Colpron, 2019). Radiogenic heat production has also been positively correlated with heat flow in the Canadian Cordillera and is therefore considered an appropriate layer to be included in the Play fairway analysis (Lewis et al., 2003). As surficial area of associated intrusive rocks is not available, a 2.5 km buffer was applied to the point measurement. This buffer was selected to remain consistent with the thermal gradient and heat flow buffers.

**Magnetotelluric –** The magnetotelluric (MT) method is a geophysical method to measure subsurface resistivity (Poux & O’Brien, 2020). MT results are dependent on rock porosity, and groundwater temperature and salinity and have been used to identify clay caps above hydrothermal reservoirs (Lautze et al., 2017; Poux & O’Brien, 2020). Clay caps typically have a resistivity below 10 ohm-m (Poux & O’Brien, 2020). Nielson et al. (2015) plan to use MT data to identify areas of hydrothermal alteration to host rock, however Lautze et al. (2017) suggest that MT data better identifies areas with high temperature and high salinity groundwater as the difference in MT results are more apparent than MT differences related to alterations. Olvera-Garcea et al. (2023) use resistivity anomaly in the MT data to identify hot fluids or rocks in the subsurface. The MT interpretation influences how it is incorporated into a play fairway analysis.

Magnetotelluric data is more valuable than gravity data as it records current conditions (Lautze et al., 2017). It should therefore be weighted greater than gravity data assuming comparable data availability.

**Gravity [gradient] –** Gravity measurements can be used to interpret rock density in the subsurface. Gravity measurements can be used to identify intrusions (dense) and contacts between host rocks with different densities (Ito et al., 2017; Lautze et al., 2017). In this sense, gravity data can be incorporated into a play fairway analysis using comparable methods to *Dike Density* or *Intrusive Rocks.* In contrast, Faulds et al. (2018) discuss the use of sharp gravity gradients combined with resistivity results to identify temperature anomalies but did not include it in the heat layer of the Play fairway analysis.

Gravity data is considered less valuable than magnetotelluric data as it represents past conditions (Lautze et al., 2017). It should therefore be weighted less than gravity data assuming comparable data availability.

**Gamma Ray Dose Rate –** Gamma ray dose rate point measurements provide information on local radiogenic isotope concentrations (Holmes et al., 2022): D = 13.2 K + 5.48eU + 2.72eTh, where Potassium (K %), Uranium (eU ppm), and Thorium (eTh ppm). Holmes et al. (2022) are the only group to incorporate this information into a play fairway analysis, but this is another metric to consider *Radiogenic Heat Production*. Holmes et al. (2022) had sufficient gamma ray dose rate data to interpolate concentrations over the study area. A buffer area could be used if data is insufficient to interpolate gamma ray dose rate.

**Basement Depth –** Holmes et al. (2022) included a map of basement depth in the heat layer of the Play fairway analysis. This depth was corrected for surface topography.

**Crustal Thickness –** An area with low crustal thickness is expected to have a higher temperature gradient compared to an area of high crustal thickness. Play fairway analyses are typically done on a regional scale where crustal thickness does not vary significantly, or any variation is associated with other geological features such as rift zones. Hinz et al. (2016) discuss the relationship between crustal thickness heat flow but do not include crustal thickness in the play fairway analysis. Holmes et al. (2022) use crustal thickness as sufficient data is available to interpolate crustal thickness over the study area.

**Surface Topography (DEM) –** Holmes et al. (2022) used surface topography to correct the basement depth estimations. Surface topography can also influence geothermal gradient as within areas of the same heat flow, geothermal isotherms are further apart in areas with high surface topography and closer together in areas with low surface topography.

**Permeability Parameters**

***Structural Setting & Faults –** Nine of the eleven geothermal Play fairway analyses reviewed include faults or structural settings into the Play fairway analysis, but the metrics used to incorporate faults vary by study. Structural settings are related to fault features such as fault terminations, fault step-overs, or fault intersections (Hinz et al., 2016; Siler et al., 2017; Lindsey et al., 2021). The exact features of interest were simplified based on study site and these were ranked based on previous research with respect to permeability and geothermal systems (Faulds et al., 2016).

For example, Lindsey et al. (2021) simplify structural setting analysis to be identified by an ellipse and include 3 settings: Pull apart (1), Intersection (0.7), and Lineaments (0.2). These ellipses denote favourable structural settings which occasional overlap and are summed by assigned value (Lindsey et al., 2021). The ellipses narrow down areas of interest on a region scale, but for local-scale research the structural settings should either be weighted and categorized based on the linear features or polygons rather than ellipses (Lindsey et al., 2021).

Where structural setting is unavailable, fault type, age, length, and distance from the fault can be used to approximate favourability. Normal faults form in extensional settings and are therefore associated with the greatest permeability favourability, followed by strike-slip faults, and thrust faults (compressional setting).

Faults can be further considered based on age and length. Young faults and surrounding fractures are expected to be more open than older faults, and the length of the fault should be mapped. Ito et al. (2017) defined a function to quantify favourability based on distance from rift or fault. The categories generated for the southwestern Yukon data were based on the function defined by Ito et al. (2017).

**Rift Zone –** Rift zones are extensional settings with elevated permeability. In regions where rift zones are well mapped, this layer provides valuable permeability information (Lautze et al., 2017). Lautze et al. (2017) note that various other features which can also be used as parameters within a permeability layer can be identified within a rift zone, notably faults and vents. Rift zones are deemed highly reliable (8/10) data sources with regards to evaluating permeability.

**Shear Stress –** Forson et al. (2015) includes maximum Coulomb shear stress and sigma 3 under the ‘stress/stain parameters applied to area surrounding fault’ subdivision of permeability in the Play fairway analysis. The Coulomb shear stress is the potential for shear fracture failure. Forson et al. (2015) is used as a proxy for fracture density within the permeability layer and assign a normalized weight of 0.206.

**Dilation Strain Rate –** Dilation strain rate at the surface is considered by Forson et al. (2015), Hinz et al. (2016), and Wang et al. (2021). The categorization of dilation strain rate is poorly defined, but a histogram of local values could be used to identify areas of high, medium, and low dilation strain or dilatation rates (see dilation tendency).

**Dilation Tendency –** Dilation tendency depends on fault geometry and are calculated based on stress models (Forson et al., 2015; Siler et al., 2017). Forson et al. (2015) include dilation tendency data for mapped and inferred faults. Siler et al. (2018) generate a histogram of maximum dilation tendency within a cell. These dilation tendencies are then grouped into high, medium, and low stress states, where a high maximum dilation tendency represents the most favourable conditions (Siler et al., 2018; Faulds et al., 2018). These are relative values, and an equivalent histogram will be unique to each study area.

**Slip Tendency –** Normal faults are consistently associated with increased permeability due to the extensional setting however, whether a strike-slip faults act as a conduit, or a barrier depends on the slip tendency. When the slip tendency is high, a strike-slip fault may act as a fluid conduit and when it is low, it acts as a barrier (Forson et al., 2015). This additional layer can therefore provide additional insight into the conditions around a strike-slip fault that fault type categorization is unable to consider. Siler et al. (2018) plot slip tendencies on a histogram and group them into high, medium, and low stress states, where a high maximum slip tendency represents the most favourable conditions. Faulds et al. (2018) groups dilation and slip tendency and tendencies of Quaternary faults.

**Fault Displacement and Displacement Gradient –** Forson et al. (2015) modelled ongoing fault displacement and extrapolated the displacement (m) across the study area. Ongoing displacement contributes to maintaining open fractures around faults. A normalized weight of 0.03 is applied within the permeability layer (Forson et al., 2015). In contrast, displacement gradient (m/m) was assigned a normalized weight of 0.175. The displacement gradient covers a smaller surface area than the modelled displacement, but both generate areas of interest around the same faults.

**Lineaments (geophysical and geomorphological) –** Lineaments identified in geophysical and geomorphological datasets were included by Olvera-García et al. (2023) in the permeability analysis as these features may represent unmapped faults. Inferred faults from geomorphological data are assigned a higher degree of confidence than those inferred from geophysical data (weight 1 and 0.8, respectively). A 300 m buffer was assigned around the lineaments. In areas where extensive fault mapping has not been completed, including lineaments as identified in remotely sensed data may provide insight into areas of increased permeability.

**Calderas –** Calderas are associated with ring fractures and zones of permeable brecciated rocks. Lindsey et al. (2021) value calderas based on the certainty of the data point, such that a caldera confirmed by peer-review and an unconfirmed or uncertain caldera are assigned values of 1 and 0.5, respectively. A 5 km buffer was placed around each mapped caldera was assigned a normalized weight of 0.19 within the permeability layer (Lindsey et al., 2021).

**Hot Springs –** Permeability is required in bedrock for hot springs to form as hot or warm groundwater rises up through preferential flow pathways. Lindsey et al. (2021) includes hot springs in the permeability layer with a 2.5 km buffer and a normalized weight of 0.17.

**Hydrothermal Alterations –** Hydrothermal alterations are evidence of fluid contact with rock which requires permeability. Lindsey et al. (2021) incorporate hydrothermal alterations into the Play fairway analysis based on the mapped spatial extent of the hydrothermal alterations (30 m resolution). This layer was then assigned a normalized weight of 0.14 in the permeability layer.

**Volcanic Vent Alignment –** Subsurface fractures are the primary drivers of permeability in bedrock. Nielson et al. (2015) suggest using the alignment of volcanic vents to predict the presence of subsurface fractures. A quantitative method to how to include volcanic vent alignment in a Play fairway analysis was not provided.

**Fluid Entries –** Poux and O’Brien (2020) identify fluid entries into wells and boreholes. This could be used on local scale geothermal favourability analyses but is not appropriate for regional analyses.

***Seismicity –** Seismicity contributes to permeability by creating and maintaining open fractures. Earthquake data have previously been used to varying extent. Wang et al. (2021) and Ito et al. (2017) only considered earthquakes shallower than 4 km and 5 km, respectively, whereas Lindsey et al. (2021) claimed earthquakes shallower than 20 km and greater than 2.5 M can aid in maintaining preferential flow pathways up to 5 km from epicenter. Due to the extensive earthquake data available for southwestern Yukon, the data was filtered to include earthquakes shallower than 20 km and greater than 2.5 M. However, in regions with more seismic activity, only considering shallow earthquakes may be more appropriate.

**Residual Gravity Anomaly –** Ito et al. (2017) used the residual gravity anomaly as an indicator of plutonic intrusions. Intrusions generate faults and fractures which increase local permeability. The residual gravity anomaly was interpolated over 3 km cells and the data is transformed based on the age of the intrusion (Ito et al., 2017).

**Magnetic Anomaly-Gradient –** Steep magnetic gradients can help identify highly fractured areas (Nielson et al., 2015). Holmes et al. (2022) transform a magnetic anomaly layer to a magnetic anomaly-gradient layer (units: degrees). Holmes et al. (2022) included the magnetic anomaly-gradient layer in the sensitivity analysis but conclude that magnetic anomaly data is low-priority data and does not have a significant control on the outcome.

**Gravity Anomaly/Gradient –** Gravity data has been used in Play fairway analyses permeability layers in numerous ways. Gravity measurements are sensitive to substrate density and can be used to identify dense intrusions (high density). Intrusions are associated with increased permeability due to the resultant faulting and fracturing related to the emplacement. Ito et al. (2017) and Lautze et al. (2017) specifically consider the residual gravity anomaly which correct the gravity anomaly for surface topography and the influence of the crust-mantle interface. Ito et al. (2017) define a formula to transform residual gravity anomaly data. The same transformation is applied to the permeability as for the transformation of gravity data for the heat layer.

Gravity gradients have also been used to identify highly fractured areas (Nielson et al., 2015; Hinz et al., 2016). Faulds et al. (2018) use gravity gradient intersections to identify subsurface fault intersections, where permeability is likely elevated due to the interaction between the faults. Despite being used extensively in Play fairway analyses permeability layer, the sensitivity report completed by Holmes et al. (2022) determined that gravity-anomaly gradients are low priority data, but the significance of gravity data may vary significantly between study sites.

**Dike Density –** Areas with high dike density may be indicative of rift systems or cordilleras. Lautze et al. (2017) consider dikes in the heat layer but exclude this data from the permeability layer. Holmes et al. (2022) include dike density in the permeability layer. Holmes et al. (2022) used units degree degree^-2^ to represent dike density like with heat parameters.

**Topographic Gradient or Elevation Change –** Topographic gradient was shown to be low priority datasets based on Holmes et al. (2022) sensitivity report. The topographic gradient did not have a significant influence on the permeability layers and can be neglected in future Play fairway analyses where more pertinent permeability related parameters are available. Wang et al. (2021) also consider including topography in the Play fairway analysis based on the relationship between elevation change and fracture pathways. The elevation change metric was deemed poorly related but was still assigned a normalized weight of 0.18 the permeability layer (Wang et al., 2021).

**Tensile Fracture Density –** All Play fairway analyses reviewed consider fractures or related features. Forson et al. (2015) differentiates between fracture types and highlight the importance of rock damage as either dilating shear or tensile fractures because these fracture types are associated with high permeability. Tensile fracture density is described qualitatively as a parameter considered in the Play fairway analysis, but it is represented in the mapped layers as Sigma 3 (*σ*_3_), the least compressive principal stress. A low or negative *σ*_3_ is associated with tensile fractures and elevated permeability. Forson et al. (2015) assigned a normalized weight of 6.5 % to the Sigma 3 map within the permeability layer.

**Fluid Availability Parameters**

**Depth to Water Table or Water Table Elevation –** Depth to the water table indicates the transition from the unsaturated to the saturated zone. This water table may not be tapped depending on the depth of the open-loop geothermal system considered but a shallow water table depth can be indicative of regional fluid availability and high fluid pressure (Hinz et al., 2016; Holmes et al., 2022; Ito et al., 2017). However, water table depth or elevation may not be representative of fluid availability at depth in areas with confining layers.

Ito et al. (2017) and Lautze et al. (2017) use the metric water table elevation. A high-water table elevation is assumed to be associated with high fluid pressure and hotter water near the surface (Ito et al., 2017). Ito et al. (2017) define a logarithmic function to associate water table elevation and fluid probability. Based on this function, any groundwater elevation greater than 500 m is assigned a fluid probability greater than 0.9 (Ito et al., 2017). This is consistent with the analysis suggested by Lautze et al. (2017) which note that fluid availability in Hawaii is not a limiting factor for geothermal resource exploration as modelled water table elevation is above the required threshold.

If water table elevation data is widely available in a study area, a natural logarithmic function related to fluid probability, or a threshold elevation could be defined. However, it is important to consider surface topography when considering using water table elevation, rather than depth to water table, in the fluid availability layer of a Play fairway analysis.

**Water Table Gradient –** Water table gradient is only suggested by Holmes et al. (2022) who define water table gradient as a low-priority metric. Water table depth or elevation provides more information about water availability and could be used independently as a fluid availability indicator in areas not restricted by confining layers.

**Groundwater Recharge –** Groundwater recharge influences fluid availability in reservoir rock. The water table is expected to be higher in an area of high groundwater recharge than an area with low groundwater recharge. Groundwater recharge can therefore be used as an indirect indication of fluid availability in areas where water table elevation data is unavailable (Lautze et al., 2017). Ito et al. (2017) pair recharge with groundwater elevation to identify areas with a combined greatest probability (high elevation and elevated recharge rate). High or low recharge rates are contextual and highly dependent on local climate. For Hawaii, Ito et al. (2017) define a normalized logarithmic function to relate recharge rates and fluid probability such that recharge rates over 0.13 cm/day provides a fluid probability greater than 0.8. This function could be adapted to be suited to different climate zones.

Some Play fairway analysis mention recharge in the context of the importance of permeability but either do not include a fluid layer or do not directly consider recharge within the fluid layer (Nielson et al., 2015; Poux & O’Brien, 2020; Lindsey et al., 2021).

**Precipitation –** Precipitation is used by Holmes et al. (2022) in the fluid availability layer as groundwater recharge was not available but highlight that precipitation is a poor predictor of fluid availability at depth and the inclusion of precipitation data in Play fairway analysis for geothermal resources is not necessary.

***Surface Waters –** Surface fresh waters have not previously been used in a Play fairway analysis for geothermal resources, but surface waters are indicative of areas where the water table reaches the surface (Witter, 1995). Though rivers and lakes have not been used as surface water indicators, the most similar fluid layer can be found in Lindsey et al. (2021) who combine surface springs with a salars and lagoon layer. A 2.5 km buffer is applied to the surface springs and the same buffer is used in the surface water layer for southwestern Yukon.

**Surface Springs –** Hot springs are used primarily in heat layers of Play fairway analyses for geothermal resources (Forson et al., 2015; Lindsey et al., 2021; Wang et al., 2021), whereas all surface springs can be used as a groundwater indicator. Surface springs are used in the fluid layers of Play fairway analysis by Lindsey et al. (2021) and Holmes et al. (2022). Lindsey et al. (2021) study site (north-western Argentina) has a high density of surface spring (thermal and non-thermal). A 2.5 km buffer is applied around surface springs. In the fluid layer, surface and hot springs were presented as present or absent and assigned normalized weights of 0.13 and 0.67, respectively.

In contrast, Holmes et al. (2022) included surface springs into the Play fairway analysis by considering the natural logarithm of spring density (number per km^2^) across the study area. Regions with high spring density are considered more favourable than regions with low spring density.

**Hydrothermal alterations –** Hydrothermal alterations are commonly used in heat and permeability layers; however Lindsey et al. (2021) also use hydrothermal alterations in the fluid layer with a normalized weight of 0.13 with a 30 m resolution of hydrothermally altered areas. Hydrothermal alterations are indicative of past presence of geothermal fluids; however it is essential to consider the age and displacement associated with the hydrothermal alterations before using them as an indicator of current fluid availability. Hydrothermal alterations may not be representative of recent conditions in all environments.

**Geothermometry –** Geothermometers are primarily used in the thermal layer of Play fairway analyses but can be used to indicate the presence of groundwater at depth. Geothermometers are therefore helpful in areas with confining geologic units for which water table elevation or depth to water table are insufficient to assess fluid circulation at depth. A geothermometer (Si) is only used in Holmes et al. (2022) fluid layer. Lindsey et al. (2021) use geothermometry to differentiate between surface springs and hot springs but not directly in the fluid model.

**Lithium & Boron Concentration –** Lithium and Boron are used by Holmes et al. (2022) as geothermometers. The lithium (mg/L) and boron (g/m^3^) concentrations is interpolated across the study area. Lithium and boron may provide useful supplementary information about geothermal fluid availability and can be used to complement the fluid layer if these measurements are widely available.

**Salars & Lagoons –** Lindsey et al. (2021) use salars and lagoons are used as a proxy for water table location. The presence of salars and lagoons are solely used in the fluid layer and are denoted based on presence (1) or absence (0) of the salar or lagoon. Lindsey et al. (2021) assign­­­ a normalized weight of 0.07 to the presence of salars and lagoons.

**Drainage Density –** Holmes et al. (2022) used a kernel density estimation technique to map drainage density based on open-source drainage polyline data. The density is defined as average channel arc per catchment solid-angle. The drainage density polylines are related to surface water features, notably rivers, which is used directly in the Play fairway analysis for southwestern Yukon.

**Magnetotelluric (resistivity) –** Magnetotelluric data is indicative of subsurface resistivity (Ωm). Ito et al. (2017) and Lautze et al. (2017) use electrical resistivity (mapped based on inversion results from a magnetotelluric study) in the fluid layer. Rock porosity, saturation, and groundwater salinity have been shown to have significant influence on magnetotelluric measurements in Hawaii (Pierce and Thomas, 2009). Lautze et al. (2017) define a fluid probability function based on resistivity (Ωm) such that probability is greater than 0.5 when resistivity is less than 750 Ωm.

Low values of electrical resistivity can indicate hot rock, magma, or a high fluid content. The presence of each of these is favourable for geothermal resources but it is essential to recall that a low electrical resistivity is not only influenced by fluid content and may be indicative of a geological feature with low resistivity, such as a clay cap.
